# Correlates of a Positive Parenting Experience in Prison

**DOI:** 10.3390/ijerph18020626

**Published:** 2021-01-13

**Authors:** Miryam Carretero-Trigo, Rodrigo J. Carcedo, Noelia Fernández-Rouco

**Affiliations:** 1 Department of Developmental and Educational Psychology, University of Salamanca, 37005 Salamanca, Spain; miryamcarreterotrigo@cop.es; 2 Department of Education, University of Cantabria, 39005 Santander, Spain; noelia.fernandezrouco@unican.es

**Keywords:** parental incarceration, caregiver, parental satisfaction, relationship with children

## Abstract

The two goals of this study were: (1) to explore the relationship of a parent’s experience in prison in combination with a range of characteristics relating to the parent in prison, to the children, and to the caregiver, and (2) to explore the role of sex and nationality in this relationship. A total of 202 parents in prison (99 men and 103 women; 106 Spanish and 96 foreigners) participated in this study. To maximize the understanding of the questions, in-person interviews were conducted to collect answers to the questionnaire. The findings particularly highlight the importance of the role of the primary caregiver in ensuring that the parent in prison has a positive parenting experience during incarceration. More specifically, the parent in prison reports a better parenting experience when they perceive the primary caregiver as a link between themselves and their children in a positive way. This finding points to the importance of intervention and research on this relationship in order to enhance parental satisfaction and the relationship between the imprisoned parent and their children, as well as the family’s resilience during parental imprisonment.

## 1. Introduction

The experience of parenting in prison is a crucial element for prisoners’ well-being and the adjustment of the family to incarceration. Few studies have addressed this issue [1], but none have used a comprehensive approach including all the significant factors found in previous research. Parenting from prison is a completely different experience from parenting in the community, outside of prison. Being outside the home and the self-redefinition that occurs within the prison environment often leads incarcerated parents to feel anxious and inadequate in their parenting role [2]. Once in prison, changes occur in family relationships and it is difficult for the imprisoned parent to participate in the lives of their children, be it financially, physically, or emotionally [3]. As a result of this, together with the associated rejection and social stigma, these families may develop physical and/or mental health problems [4,5].

From a family resilience perspective [6], resilience, conceptualized as the “capacity of a dynamic system to adapt successfully to disturbances that threaten system function, viability, or development” [7] (p. 6), is extended to include family functioning with a special focus on the relationships between the various members [8]. Parental satisfaction and satisfaction with the relationship prisoners have with their children are two necessary indicators of the extent of a family’s resilience. Previous research showed that improvements in resilience competences through the implementation of parenting programs are associated with higher parental satisfaction [9].

Parenting satisfaction is defined as a sense of pleasure and fulfilment in relation to the parenting role [10]. The satisfaction of parents with regard to their relationship with their children refers to the fulfilment of one’s wishes, expectations, or needs, or the pleasure derived from their current relationship with their children. This aspect is more specific, and it is included in the evaluation of the parenting role, which it is crucial to evaluate separately because it is important for inmates’ well-being [11]. Contact and communication with children are two important inmate needs that are, unfortunately, difficult to fulfill during incarceration.

Although it is only one perspective (the views of children and main caregivers are also central), the point of view of incarcerated parents is important in understanding the family process. Placing a special emphasis on relationships, from the experience of the parent in prison, we have to consider the existence of factors that could lead to greater parental satisfaction in terms of parenting and in terms of their relationship with their children. In this regard, we should not only consider a range of variables relating to the parent in prison, but also their own experience with their children and with the primary caregiver during their time in prison.

In relation to the parent in prison, previous studies have mentioned the possible relevance of different sociodemographic aspects such as sex, nationality, educational level, and the partner’s relationship. The sex of the imprisoned parent impacts the children through the continuity of care and the frequency and type of contact established [12,13,14,15,16,17]. Men may have a more positive experience of parenthood in prison insofar as their children continue to receive the same care as they had been receiving prior to imprisonment. This facilitates the need to maintain family unity and communications. In the case of women, they are more likely to experience a more negative experience of parenthood as they must accept that they are no longer the primary caregiver, which they may have been until the time of their imprisonment [3,12,13,18,19]. The same phenomenon can be observed in the case of nationality: about 20% of Spaniards have no communication with their relatives and this percentage doubles for foreigners, reaching 40%. In the case of visits, whereas 57% of Spaniards receive visitors, 57% of foreigners never receive a single visitor during their incarceration [20]. With respect to the relationship sustained with children, an aspect of the parental experience included in this research, some studies found that foreign nationals in prison experience a poorer relationship with their children. This negative assessment of the relationship with their children is accounted for by the lack of contact with their children due to the separation distance [21]. Both of these variables, which are explored in this research, could have direct and moderating effects on parental satisfaction and the relationship between children and their imprisoned parents.

Similarly, some studies have found an association between the level of education and the mother’s ability (no data in the case of fathers) to respond to the needs of their children [22,23], with the level of education in prison tending to be lower than that of the non-prison population [24,25,26]. Relationships are another significant variable for imprisoned parents. In the case of men, there is a close association between marital status and contact with one’s children, with married prisoners receiving higher numbers of visits from their children [27]. This frequency decreases, however, in the case of couples who begin the process of separating [28]. The results of this work with imprisoned parents therefore suggest that the partner’s bond can be a facilitator of the parenting experience. In summary, all these sociodemographic characteristics could be associated with a positive experience of parenting during incarceration; however, it is unknown whether these variables would still be significant when all of them are analyzed together in conjunction with other penitentiary, children, and caregiver aspects.

The relationship between an imprisoned parent and their family might also be affected by the prisoner’s situation in prison. Thus, relationships among family members might be facilitated by the application of certain prison benefits such as furloughs, access to a minimum-security level or parole, among others, or hindered by a lengthy sentence or a more restrictive classification [21,29,30]. In general, people tend to use prison furlough where possible [20,31]. Although only a small percentage are eligible for these penitentiary benefits, inmates typically make good use of them [20].

In the case of variables associated with children, a larger number of children has been associated with lower levels of parental satisfaction [32,33] due to higher levels of stress [34]. The average number of children of women in prison is greater than that of men [35]. The age of the children is also associated with different needs and different forms of contact [36,37]. In the case of younger children, visits represent the most effective form of communication between the imprisoned parent and the child, as letters or telephone calls are not adapted to the child’s abilities and needs [38]. In this sense, parents with younger children may find the experience of parenthood in prison more difficult, as the opportunities for bonding through visits are scarce [15].

One of the main concerns of families is whether or not to communicate to the child the fact that their parent has been imprisoned. Parental imprisonment could negatively impact short-term emotional well-being as well as long-term health and social prospects [39,40], although the inability to talk about or obtain information is also associated with greater levels of distress [16]. This, in turn, is a major concern for families and the imprisoned parent [30,41]. Thus, parents whose children are unaware of the situation could view the experience of parenthood as being worse, as they are concerned with how to explain what happened and are concerned about their children’s distress over their absence.

For the imprisoned parent, the psychological distress experienced may be related to the extreme difficulties they face in remaining in contact with their children and families [42]. For this reason, communication between these children and their parents has been linked to their involvement in their children’s lives and the post-release relationship [43]. For children, visits are often reassuring in relation to their parents’ well-being, reducing negative feelings resulting from separation and helping them to overcome some of the negative effects associated with a parent’s absence and the disruption of the family unit [44]. In terms of the type and frequency of contact, women have more contact with their children by telephone and through letters compared to men [14], whereas men receive more visits from their children than women [13]. By maintaining more contact with their children, parents are more likely to be involved in their children’s lives and tend to have a better perception of their own parenting.

Many imprisoned parents wish to return to their families, but fear that they will not be able to meet the expectations and needs, especially when they have not maintained contact during their stay in prison. These parents are often unable to make decisions about contact with their children and are at the mercy of the caregivers’ decisions. The figure of the caregiver and how they act as a link to the parent in prison could therefore be of particular relevance to the overall experience of parenthood in prison. The most critical change for these children relates to the role of the caregiver in the absence of the imprisoned parent. Some studies link the welfare of children and the current figure of the caregiver [45] to their ability to care for and promote child development [46], suggesting that the children of male prisoners suffer less stress than the children of female prisoners [47]. This may be due to the children remaining mostly in the care of their mothers, as they were before their father entered prison, whereas the children of women in prison are mostly in the care of their grandmothers [13]. Similarly, the relationship between the caregiver and the imprisoned parent, or the caregiver’s nexus function, is important to understanding parenthood in prison and the return to family life after imprisonment [48,49]. Some caregivers support the imprisoned parent/child relationship by encouraging contact; others hinder or limit such contact. Positive caregiver–parent relationships have been associated with increased frequency of contact and greater emotional stability in children [14,45,50,51,52], but it is unknown whether a significant relationship exists with parenting satisfaction. In addition, if the caregiver fails to fulfil their nurturing role due to a poor relationship with the imprisoned parent, the consequences for the imprisoned parent may be a lack of involvement in their children’s lives and a consequent loss of authority over their children [53,54].

To date, no studies have investigated the association between the parent, the children, and the caregiver’s characteristics alongside the parenting experience of prison inmates. These associations might be affected by the sex and nationality of the inmates. In this study, we addressed these relationships and their moderating effects, which are our main contributions to understanding the parenting experience in prison. As such, we explored the prison parenting experience addressing two questions: (1) What is the relationship between the socio-demographic and prison characteristics of the parent and the variables related to the children and the caregiver, and the parenting experience of prison inmates? (2) What is the moderating role of sex and nationality in this relationship?

## 2. Materials and Methods

### 2.1. Participants

A total of 226 inmates were interviewed, 14 of whom were excluded from the study as they were Spanish citizens residing abroad or foreigners residing in Spain. To homogenize the sample in relation to this variable, we chose to only include Spaniards residing in Spain and foreigners residing abroad at the time of the interview.

The total sample consisted of 202 interviewees, distributed as follows: 51% (*n* = 103) women and 49% (*n* = 99) men; 52.5% (*n* = 106) were Spanish and 47.5% (*n* = 96) were foreigners. Of these 99 men, 55 (55.6%) were Spanish and 44 (44.4%) were foreigners. In the case of women, 51 (49.5%) were Spanish and 52 (50.5%) were foreigners. The parents interviewed had an average age of 34.60 years (SD = 7.72), with the oldest participant being 57 and the youngest 19 years. Of the total sample, 73.26% had completed primary education, or its equivalent in the country of origin. Finally, 57.92% of the people interviewed were in a relationship when the study was conducted.

The sample was drafted via the various activities being carried out by a non-governmental organization (NGO) in a prison in western Spain. This NGO organizes activities in all prison units, so the vast majority of parents in prison with children deemed to be minors were invited. The interviewer was in charge of the work conducted by the NGO in the aforementioned prison and hence had already established a relationship with the participants, an aspect that helped to ensure the veracity of the answers obtained. As such, none of the invited individuals declined to participate in this research, despite being informed of the option not to participate or to withdraw at any time.

By cross-referencing the sex (men/women) and nationality (Spanish/foreign) variables, four study groups were generated (Spanish men, foreign men, Spanish women, and foreign women). An attempt was made to select 50 people from each group or profile so that, when that number was reached, no further interviews were conducted with individuals from that group. The inclusion criteria for forming the sample were:

(1) Being the parent of a minor: only parents with children deemed to be minors were considered, as older children do not require an adult to interact with their mother or father in prison.
(2) Having been in prison for at least six months: this measure was chosen in an attempt to prevent the anticipated initial effect of imprisonment on the mental health of the imprisoned parent.
(3) Having made attempts to maintain a relationship with their children in the previous six months: as the object of the study is the experience of parenthood in prison, the attempt to maintain a relationship or connection with one’s children as a means of pursuing this end was a requirement in this study.
(4) Fluency in Spanish: although this criterion is likely to reduce the representativeness of the sample, it guarantees a more reliable response as it would be easier to transmit and understand the dialogue of the interview.

The following provides a description of the variables specific to this study: persons involved, parental experience, and mental health, as well as the instruments of measurement for each of them.

### 2.2. Measures

#### 2.2.1. Incarcerated Parent Variables

##### Sociodemographic and Penitentiary Variables

Sex (0, men; 1, women), nationality (0, Spaniards; 1, foreigners), age, partner status (0, no partner; 1, with a partner), and educational level (0, not having completed primary education; 1, having completed primary education) were included as sociodemographic variables in this study. In addition, a set of penitentiary variables were added: penitentiary situation (0, convicted; 1, preventive), number of days of prison furlough during the last six months, the total time spent in prison, having a paid job in prison (0, no; 1, yes), and sending money to the children (0, no; 1, yes).

#### 2.2.2. Variables Concerning the Children

These variables include the number of children each inmate had, the average age of all the children of each parent interviewed, the child knowing that their parent is in prison (0, no; 1, yes), and the number of times the inmates have communicated by mail, phone and/or visits with their children.

#### 2.2.3. Caregiver Variables

**Figure 1:**
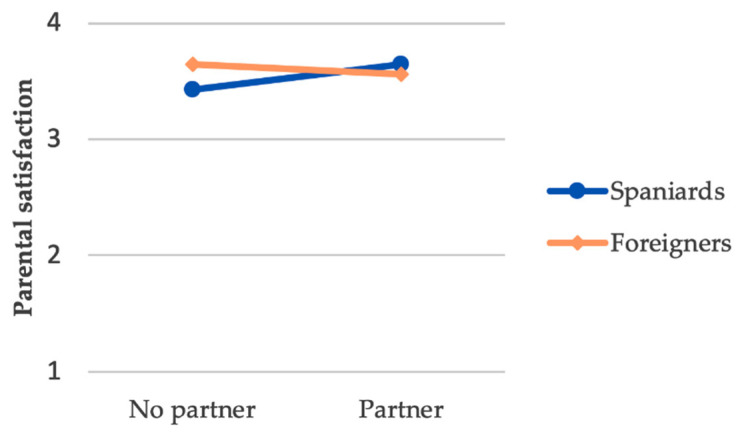
Partner*nationality interaction for parental satisfaction.

Two more variables were considered for caregivers. The first was the figure who assumed responsibility as caregiver of the children. This variable was collected via a question that reflected 9 options with different possible caregiver figures (1, other parent; 2, grandparents; 3, paternal grandparents; 4, other relatives; 5, older siblings; 6, adoptive family; 7, a friend; 8, juvenile facility; 9, other). All the answers referenced either the other parent or one of the grandparents; thus, this variable was grouped into just 2 categories: 0, grandparents; 1, other parent.

The second variable was the nexus function of the caregiver between the imprisoned parent and the children. A single 4-item ad hoc scale was used to determine to what extent (1) the relationship between the imprisoned parent and the primary caregiver was good, (2) the primary caregiver supported the relationship between the imprisoned parent and their children, (3) the primary caregiver allowed the imprisoned parent to participate in the care and education of their children, and (4) the primary caregiver allowed the imprisoned parent to participate in the day-to-day life of their children. The response format was a Likert-type scale, ranging from 1, indicating strongly disagree, to 5, which indicated strongly agree. The scale presented a Cronbach’s α reliability of 0.94.

#### 2.2.4. Parenting Experience: Parental Satisfaction and Relationship with Children

Parenting experience is defined as the reciprocal influence and relationship between parents and children and the judgments concerning the value of the parenting experience [55]. Two main dimensions of this experience are highlighted within this concept: parental satisfaction and relationship with children. Two subscales from Guidubaldi and Cleminshaw’s Parental Satisfaction scale [55], namely parental satisfaction and relationships with children, were used in this study. Both subscales contained 10 Likert-type items and the same response format (from 1, indicating strongly disagree, to 4, indicating strongly agree), and presented good reliability (Cronbach’s α = 73 for parental satisfaction and Cronbach’s α = 77 for the relationship with children). These measures were selected because they had already been used successfully in the prison population [56].

### 2.3. Procedure

All the interviews were conducted in a single session, in a small office located within the different blocks within which the participants of this study resided. As explained above, the interviewer had already established a relationship with the participants due to their work for the NGO involved in this prison. The methodology of the study was explained to them, guaranteeing total confidentiality and respect for their anonymity. All the research processes were conducted in accordance with the ethical standards of the American Psychological Association and all the norms of the Declaration of Helsinki were respected in this investigation. Once the proposal was provided, they were prompted to sign the informed consent agreement and the interview was subsequently conducted. The interview was conducted in a single session, lasting an average of approximately 60 min, and ended by thanking the interviewee for their collaboration.

### 2.4. Data Analysis

The data obtained through the semi-structured interview were processed with the statistical package SPSS 25.0 (IBM Corp., Armonk, NY, USA). To study the incidence of the variables of the persons involved in the experience of parenthood, bivariate correlations (Pearson’s, point-biserial, and phi coefficients) and multiple hierarchical regression analyses were used. For the analysis of the interactions, the macro PROCESS 3.5, developed by Hayes [57], and the pick-a-point approach were used [58,59,60].

## 3. Results

The following results are the correlations between all the study variables for the total sample, and separately for men, women, Spaniards, and foreigners (Table 1, Table 2 and Table 3).

Once the results obtained with the bivariate correlations were reviewed, the joint effect of the predictors showing significant correlations with the two criterion variables of the experience of parenthood (parental satisfaction and relationship with the children) was analyzed, studying the possible moderating effect of the sex and nationality variables. To this end, two hierarchical multiple regression analyses were performed. The predictors included in these analyses were those that showed significant correlations with the criterion variables of the experience of parenthood. We decided that the predictors would be introduced through different blocks of variables.

Consequently, the first block comprised the sociodemographic variables of the parent in prison, the second block added the prison variables, the third block added the children’s variables, the fourth block included the caregiver variables, the fifth block added the moderating variables, and the sixth block included the interactions of the different predictors with the sex and nationality variables.

In the block of interactions (interaction model), the variables that correlated significantly with the criterion variable for one of the sexes but not for the other, or for one of the nationality groups but not for the other, were added. We also added the predictor variables that correlated significantly with the criterion variable in both groups, which included the sex and nationality variables, albeit with opposite signs.

Finally, following the principle of parsimony, if the contribution of the block of interactions was not significant for explaining each variable, only one main effects model was chosen. Conversely, if the percentage of variance explained by the model of interactions was statistically significant, the significant interactions were incorporated into the final model.

For the parental satisfaction variable, the model of interactions proved to be significant, so this model was chosen as the final model. Likewise, following the principle of parsimony, only the main effects and significant interactions were introduced into the final model (Table 4). The Variance Inflate Factor (VIF) was lower than 10 for all of the main effects and their interactions [61], ranging from 1.04 to 2.84.

When analyzing the semipartial squared correlations, the results indicated that the nexus function of the caregiver was the variable that explained the most variance (*R*^2^ = 0.042), followed by the partner × nationality interaction (*R*^2^ = 0.035) and the sending of money (*R*^2^ = 0.027). Here, a higher value for the caregiver’s nexus function was associated with higher parental satisfaction. Conversely, sending money to children was associated with lower levels of parental satisfaction.

With respect to the significant partner × nationality interaction (Δ*R*^2^ = 0.075 *), having or not having a partner was relevant only for the group of Spaniards (*B* = 0.217, *p* = 0.003); thus, among Spaniards, having a partner was associated with a higher score on the parental satisfaction scale (Figure 1).

Regarding the relationship with children variable, the model of interactions was again significant, so this model was selected. Following the principle of parsimony, only the main effects and significant interactions were introduced into the final model (Table 5). VIF was lower than 10 for all the main effects and interactions, ranging from 1.14 to 6.76. The analyses of the semipartial squared correlations revealed that the nexus function of the caregiver was the variable explaining the most variance (*R*^2^ = 0.282), followed by partner × nationality interactions (*R*^2^ = 0.027), furloughs × sex (*R*^2^ = 0.019), and number of children × nationality (*R*^2^ = 0.014). Again, a greater value for the nexus function of the caregiver was associated with greater parental satisfaction. For the analysis of interactions, the partner × nationality, furloughs × sex, and number of children × nationality interactions proved to be significant.

**Figure 2:**
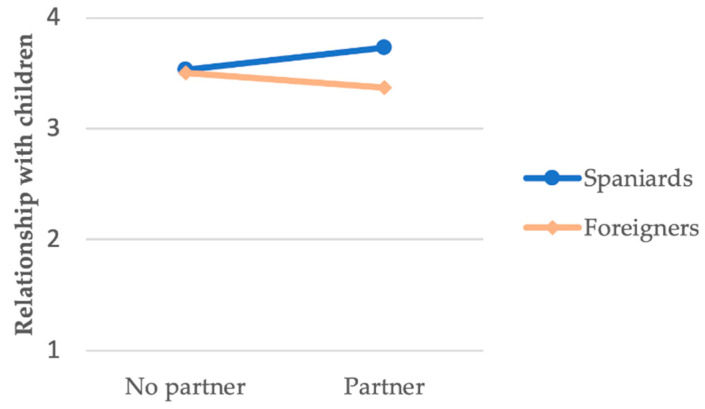
Partner*nationality interaction for relationship with children.

In the partner × nationality interaction, having or not having a partner only proved significant in the case of Spaniards (*B* = 0.203, *p* = 0.010), whereby having a partner was associated with a greater appreciation of the relationship maintained with their children, while no relationship was observed for the group of foreigners (Figure 2).

**Figure 3:**
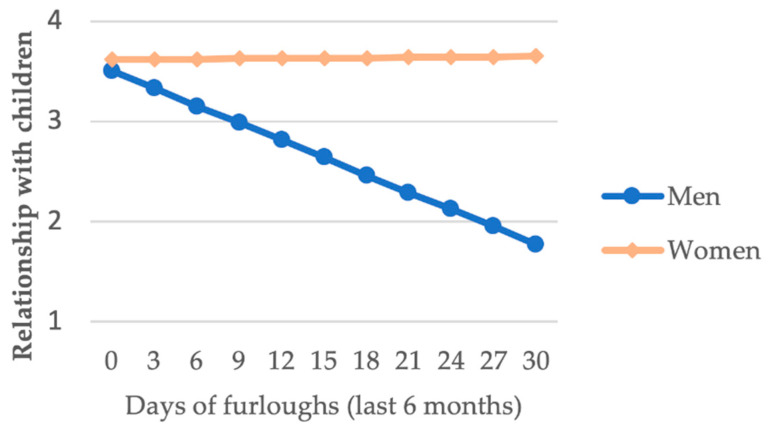
Days of furlough*sex interaction for relationship with children.

In the furloughs × sex interaction, taking furlough was only relevant in the case of men (*B* = –0.057, *p* = 0.004). Thus, in the case of men, having enjoyed more furlough days was associated with a lower appreciation of the relationship maintained with their children, whereas this relationship was not observed in the group of women (Figure 3).

**Figure 4:**
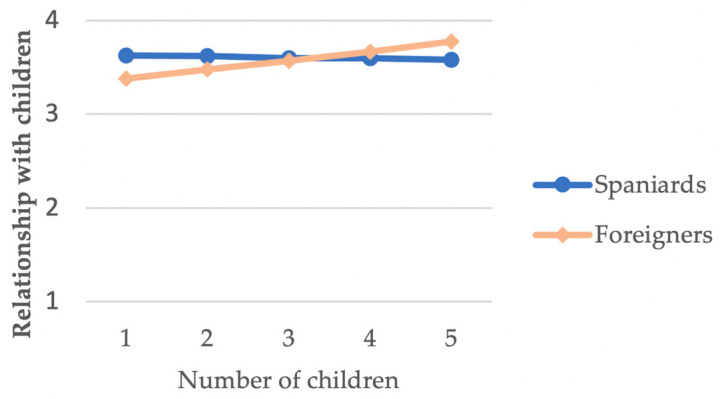
Number of children*nationality interaction for relationship with children.

Finally, in the interaction of the number of children × nationality, having more or less children was only relevant in the case of foreigners (*B* = 0.098, *p* = 0.006). Having a larger number of children was associated with a better assessment of the relationship maintained with them among the group of foreigners, with no relationship observed among the group of Spaniards (Figure 4).

## 4. Discussion

In this study, we explored the relationship between a parent’s experience in prison and a range of characteristics (socio-demographic and prison) related to the parent in prison, along with characteristics related to the children and to the caregiver. Finally, the role of sex and nationality in this relationship was also examined.

From the results of this study, we found that the nexus function of the caregiver appears in the two criterion variables that define the experience of parenting (parental satisfaction and relationship with children) as the predictor with the greatest explanatory power. This result agrees with that of Berger [62], suggesting that caregivers are responsible for maintaining the mental bond and communication between parents and children [42] and/or facilitating family reunification after release [48]. Similarly, Loper and Tuerk [63] confirmed the need to be aware of the alliance between parents or between parents and caregivers when designing interventions for parents in prison. This is particularly important because they have the capacity to facilitate and increase the frequency of contact between imprisoned parents and their children [14,45,50,51]. Accordingly, the possibilities of using social media, such as videoconference calls, should be considered to increase opportunities for communication between parents and children [64]. This family-centered strategy seems to benefit not only the relationship, but also improves the situation for inmates in the prison setting, reducing misconduct in prison [65].

These data coincide with the systemic models that suggest that a change in part of a system entails the reorganization in terms of roles and functions of the rest of the system, modifying the resulting experiences [66]. When a parent enters prison, the family system needs to define who is going to be the caregiver of the children in order to ensure their upbringing. This caregiver plays a key role in making decisions concerning the children, acting as a mediator between the imprisoned parent and the children. Likewise, this result highlights the importance of family relationships as a central element in increasing family resilience to the overall situation of a parent entering prison [6].

By analyzing each criterion variable, we observed that, in the case of parental satisfaction, the caregiver’s nexus function (caregiver variable), the sending of money (parent variable), and the partner × nationality interaction accounted for 14.6% of the variance.

Studies that support the idea that the caregiver’s nexus function is associated with levels of parental satisfaction have focused primarily on the application and evaluation of programs for parents in prison [67,68,69,70]. These programs have been found to help strengthen the relationship between the incarcerated parent and their children’s caregiver, while improving parenting skills and increasing levels of parental satisfaction [9,41,63,71,72,73]. Consequently, interventions aimed at improving the parenting competences of prison inmates would be helpful in strengthening the parent–children relationship and parenting satisfaction [9].

A negative relationship was observed between parental satisfaction and sending money: parents who sent money to their children, to a greater extent, experienced lower levels of parental satisfaction. Some studies partially supported this idea of the inverse relationship between sending money and parental satisfaction, suggesting that parents with higher levels of satisfaction are less concerned about their children’s financial situation and more focused on their emotional needs [69]. Interventions with parents in prison should promote their active participation in their children’s lives [74] and their involvement in their children’s emotional needs, but not only through financial support.

The partner × nationality interaction was significantly associated with parental satisfaction for Spaniards who had the support of their partner, as they experienced the highest levels of parental satisfaction. Spaniards experience greater closeness and availability from their partner than foreigners [21]. The greater availability and accessibility of the partner may translate into decreased levels of loneliness [75], which may, in turn, lead to increased levels of parental satisfaction. Some studies that support these results argued that a relationship exists between the levels of loneliness experienced and parental satisfaction [76]. As such, the partner is able to help maintain the bond and hope for reunification, thus improving parental satisfaction levels. Conversely, when divorce or separation proceedings are initiated within a couple, maintaining a bond with their children becomes much more complex for the imprisoned parent [28]. Policy and programming support could help justice-involved couples maintain contact during incarceration [77,78,79,80] and assist with strengthening couples’ communication as they prepare for the partner’s return to the community [81].

In the context of parenting, 47.2% of the variance of the relationship with children variable was explained by the nexus function of the caregiver, and the interactions of partner × nationality, furloughs × sex, and number of children × nationality. This suggests that a higher appreciation of the caregiver’s nexus function is associated with an improved relationship status with the children. This finding is in line with results reported in previous research [14,36,82,83,84,85]. The studies cited suggest that most parents understand that their child’s caregiver performs actions to foster the relationship between the parent and child, such as facilitating contact [83,86]. As mentioned above, these findings highlight the importance of adopting a systemic perspective with regard to explaining the new roles generated by parental imprisonment [66], while referring to relationships that can generate increased family resilience [6].

The first interaction, partner × nationality, indicates that imprisoned Spanish parents who have a partner more highly value the relationship they have with their children. There are no known studies that either support or oppose these findings. However, this result was repeated. Both the relationship with the children and parental satisfaction were associated with the interaction between partner and nationality. A possible explanation for this result could be that Spaniards have more opportunities to access their partner than foreigners. In this sense, it is easier for them to perceive such partners as supportive, facilitating their relationship with their children. Nesmith and Ruhland [85] suggested that the actions and feelings that women express toward imprisoned men affect the attitudes that children have toward their fathers. If the attitudes of the partner toward the imprisoned parent are positive, according to the work cited, the children will show a positive attitude toward the absent parent, thus improving the relationship between the imprisoned parent and their child. Having a partner was associated with a greater appreciation of the relationship maintained with one’s children and higher parental satisfaction in Spaniards, probably because they had greater access to their children than foreign inmates. Even so, this interaction is difficult to interpret because, in this study, no differentiation was made as to whether the partner was the other parent and/or the current caretaker of the children, amongst other reasons, as indicated in the limitations section of this study. Regardless, as we mentioned previously, the use of social media could help increase the opportunities for communication between partners [64]. This could have special importance for inmates whose parents live far away from the prison, as occurs in the case of foreign inmates.

The furloughs × sex interaction suggested that men who take more furlough days consider the relationship with their children to be worse. The explanation for this result may be related to the expectations of the imprisoned parent, who maintains an unrealistic image of the situation the family is experiencing, and the newly established dynamics, tending to idealize the relationship [87,88]. Some studies have suggested that men in prison have more optimistic expectations than their children’s mothers regarding the relationship with their children and their eventual reunion [83,89]. The possibility of living with children while on furlough can lead to a clash between expectations and actual encounters with regard to their skills and levels of parental authority [90] and in relation to the perceived discomfort of the children at having to live with someone who has been absent for a considerable period of time [91].

Unrealistic expectations regarding the family are a further consequence of the process of imprisonment, which is closely linked to time spent in prison. Increased time in prison leads to the idealization of the family and a discordance with encountered reality [92,93]. More time in prison distances people from the dynamics and reality of family life [88]. The data in this paper indicated that Spanish men serve more time in prison. As a result, men may have expectations that are less attuned to the actual family situation due to their longer sentences. If we add to this the fact that men receive fewer furloughs than women [20], these men may be less likely to contrast their expectations of the relationship with their children with the reality they are experiencing. Therefore, the impact of the furlough is more likely to result in a more negative assessment of their relationship with their children. Preparing fathers in prison for furloughs could provide new opportunities for intervention with them. Managing idealized expectations about furlough with the family and training fathers to develop communication skills that they can apply when communicating with their families may be two important aspects that could make this penitentiary benefit a success for the inmate.

This relationship between the number of furlough days taken and the assessment of the relationship with one’ s children is absent in women due to factors relating to life prior to entry into prison. As noted above, these women are often the primary and/or sole caregivers for their children prior to entry into prison, whereas men tend to play more peripheral roles [39]. In this sense, women are likely to have more insight and a more accurate idea of their children’s situation. The expectations of these women may be more in tune with the current family reality. In relation to these data, people in prison need to prepare for their re-entry into society, as suggested by Kazura [94], particularly as the idea of a family reunion is difficult for them to address.

The last interaction, number of children × nationality, suggested that foreigners who have more children attach greater value to their relationship with them. Previous studies have found that an increased number of children is associated with increased parental stress and decreased parental satisfaction, but this is not the case when assessing the relationships they have with their children [32,33,34,95,96]. The results of this study, however, suggest that, among foreigners, a higher number of children is associated with a more favorable assessment of parents’ relationships with their children. No studies have been found to support this result. To understand these data, it may be necessary to ascertain the incidence of cultural variables that define the experience of parenthood in one aspect or another and, therefore, the relationship with the children, as parenthood is an experience that varies according to the culture. Speculatively, perhaps having more children may encourage at least some inmates to maintain closer contact with their children during their time in prison, which may, in turn, be reflected in a more favorable assessment of this relationship.

### Limitations and Future Lines of Research

Although this work offers interesting results, it is not free of limitations. Being a cross-sectional design, the information is limited to a single point of measurement, preventing the establishment of causal relationships. It is possible that circularity exists between the studied variables. Nevertheless, previous studies and the theoretical models exposed in this work indicate that the relationship between the variables follows the same direction. Future studies should involve a longitudinal study to establish some causal relationships between the experience of parenthood and the different predictors.

Another limitation of this study is centered on the figures of the partner, the other parent, and the caregiver. Although it was clearly established whether or not the other parent was the caregiver of the children, the role played by the partner in these relationships, apart from their role as a partner, is unclear. Whether the current partner of the imprisoned parent acts as the other parent of the children and therefore the primary caregiver is uncertain. In this sense, future studies should focus on the role played by the partner throughout the framework of parenting. It is necessary to comprehend what relationship this partner has with respect to the children, namely understanding if they are the other parent of the children and/or the present caregiver. With regard to children, it would be beneficial clarify the effect of a higher or lower number of children on the relationship between imprisoned parents and their children. Similarly, it would be helpful to identify the key issues discussed by these parents, their children, and their caregivers over the telephone, as this is the most common form of communication and the one that most predicts the experience of parenthood in prison. Following this, forms of social media were not explored in this research regarding the contact between parents in prison and children outside because this type of communication with the family was only allowed in exceptional situations. However, this resource was used more frequently following the COVID-19 pandemic. Future research should address the effect of this type of communication from prison on family relationships.

Finally, and based on the primary outcome of this work, a line of study concerning the caregivers of these children should be initiated. More knowledge is needed in terms of the needs and resources that these people have when facing this situation. The impact it has on their lives and their mental health is also relevant. Furthermore, the skills and characteristics of caregivers who are able to act as a bridge between the imprisoned parents and their children should be identified, as should the difficulties encountered by caregivers who are unable to fulfil this bridging function.

## 5. Conclusions

Despite the various outstanding research issues, this paper provides new and relevant information by exploring the aspects that are most relevant to parental satisfaction and the relationship between imprisoned persons with their children, focusing on the imprisoned parents rather than on other family members, as has been more common in previous research. Similarly, we considered the role of the caregiver, which appears to be a central element in the parenting experience of people in prison, with the relationship established with the parent in prison being a key aspect for clinical and socio-educational intervention. In this sense, the work with the caregiver becomes an essential element of intervention work, as the caregiver acts as a mediator in the relationship between children and parents in prison. Promoting quality communication and emotional management strategies among these agents will therefore allow for the optimal development and well-being of the various parties involved.

## Figures and Tables

**Table 1:** Pearson correlations between the parenting experience (parental satisfaction and relationship with children) and the variables concerning the imprisoned parent, the children and the caregiver for the total sample.

	1	2	3	4	5	6	7	8	9	10	11	12	13	14	15	16	17	18	19	20
1. Parental satisfaction	−																			
2. Relationship with children	0.53 **	-																		
3. Sex (1 = women)	0.04	0.14 *	-																	
4. Nationality (1 = foreigner)	0.05	0.12	0.06	-																
5. Age	−0.09	−0.02	−0.08	−0.16 *	-															
6. Partner (1 = yes)	0.13	0.16 *	−0.15 *	−0.09	0.04	-														
7. Primary Education (1 = yes)	0.00	−0.04	−0.07	0.32 **	−0.08	−0.19 **	-													
8. Penitentiary situation (1 = preventive)	0.13	0.14 *	−0.02	0.06	−0.13	0.10	0.12	-												
9. Time in prison	−0.13	−0.23 **	−0.15 *	−0.17 *	0.22 **	−0.02	−0.07	−0.32 **	-											
10. Furloughs	−0.00	0.0.02	0.15 *	−0.02	0.07	0.11	−0.05	−0.13	0.10	-										
11. Remunerated job (1 = paid-employment)	−0.08	−0.04	0.09	0.22 **	0.07	0.02	0.04	−0.24 **	0.16 *	0.01	-									
12. Sent money to their children (1 = yes)	−0.14 *	0.00	0.11	0.19 **	0.07	0.03	0.07	−0.16 *	0.13	−0.01	0.70 **	-								
13. Number of children	0.12	0.12	−0.03	−0.00	−0.11	0.18 **	−0.23 **	0.07	−0.06	−0.04	−0.12	−0.04	-							
14. Age of children	0.02	0.08	0.16 *	0.00	0.58 **	−0.01	−0.13 *	−0.17 *	0.21 **	0.15 *	0.09	0.06	−0.03	-						
15. Children know parent is in prison (1 = yes)	0.17 *	0.19 **	0.03	−0.20 **	0.26 **	0.14 *	−0.26 **	0.00	0.07	0.02	−0.10	−0.08	0.16 *	0.35 **	-					
16. Letters	0.13	0.18 **	−0.08	−0.14 *	0.10	0.07	−0.08	0.03	−0.05	−0.06	−0.00	0.03	0.01	−0.01	0.03	-				
17. Phone	0.19 **	0.32 **	0.04	−0.07	−0.12	0.17 *	−0.02	0.04	−0.04	0.05	0.11	0.02	0.05	−0.08	0.15 *	0.12	-			
18. Visits	0.12	0.20 **	0.00	−0.39 **	−0.03	0.22 **	−0.18 **	0.06	−0.00	0.04	−0.01	−0.09	0.02	0.01	0.26 **	0.18 **	0.36 **	-		
19. Figure of caregiver (1 = other parent)	−0.01	−0.07	−0.44 **	−0.06	0.12	0.06	0.03	0.02	0.08	−0.18 *	−0.07	−0.10	0.01	−0.05	0.02	0.11	−0.12	0.01	-	
20. Nexus function	0.28 **	0.62 **	0.02	0.19 **	−0.05	0.24 **	−0.02	0.12	−0.10	0.09	0.04	0.05	0.08	0.01	0.09	0.09	0.42 **	0.21 **	−0.09	-

* *p* < 0.05, ** *p* < 0.01. Note: Pearson correlations were used between two continuous variables, point-biserial correlations between a categorical and a continuous variable, and phi coefficients between two categorical and dichotomous variables.

**Table 2:** Pearson correlations between the parenting experience (parental satisfaction and relationship with children) and the variables concerning the imprisoned parent, the children and the caregiver for men and women.

	1	2	3	4	5	6	7	8	9	10	11	12	13	14	15	16	17	18
1. Parental satisfaction	−	0.60 **	−0.11	0.10	0.03	0.12	−0.17	−0.05	−0.09	−0.15	0.13	0.07	0.25 **	0.20 *	0.21 *	0.03	0.05	0.26 **
2. Relationship with children	0.47 **	−	−0.09	0.22 *	−0.02	0.11	−0.16	0.09	−0.04	0.06	0.13	0.11	0.23 *	0.19	0.34 **	0.20 *	0.22	0.62 **
3. Age	−0.06	0.05	−	0.06	−0.18	−0.28 **	0.26 **	0.09	0.03	−0.04	−0.06	0.59 **	0.28 **	0.10	−0.14	0.03	0.11	−0.21 *
4. Partner (1 = yes)	0.18	0.14	−0.00	−	−0.31 **	−0.03	0.04	0.17	0.08	0.06	0.21 *	0.13	0.20 *	0.15	0.17	0.26 **	−0.05	0.18
5. Primary Education (1 = yes)	−0.02	−0.04	0.01	−0.09	−	0.09	−0.11	−0.05	0.01	0.05	−0.37 **	−0.16	−0.26 **	−0.11	0.05	−0.18	−0.06	0.08
6. Penitentiary situation (1 = preventive)	0.14	0.18	0.00	0.04	0.15	−	−0.31 **	−0.16	−0.26 **	−0.17	0.07	−0.31 **	−0.04	0.13	0.15	0.08	−0.05	0.14
7. Time in prison	−0.11	−0.25 *	0.19	−0.11	−0.07	−0.35 **	−	0.12	0.33 **	0.16	−0.07	0.21 *	0.01	−0.09	−0.07	0.07	−0.24 *	−0.01
8. Furloughs	0.10	−0.21 *	0.09	0.08	−0.02	−0.11	0.21 *	−	0.00	−0.04	−0.07	0.18	0.01	−0.06	0.09	0.09	−0.17	0.14
9. Paid job (1 = paid−employment)	−0.08	−0.06	0.13	−0.02	0.11	−0.22 *	0.09	0.02	−	0.66 **	−0.09	0.16	−0.12	−0.13	0.13	−0.03	−0.11	0.03
10. Sent money to their children (1 = yes)	−0.14	−0.09	0.23 *	0.04	0.12	−0.16	0.15	0.01	0.74 **	−	0.03	0.05	−0.15	−0.09	0.01	−0.16	−0.03	0.14
11. Number of children	0.11	0.13	−0.16	0.14	−0.09	0.08	−0.07	0.03	−0.15	−0.12	−	−0.11	0.22 *	0.23 *	0.07	0.12	0.10	−0.01
12. Age of children	−0.04	0.02	0.67 **	−0.11	−0.08	−0.02	0.28 **	0.04	−0.02	0.03	0.04	−	0.44 **	−0.01	−0.06	0.14	0.07	0.00
13. Children know parent is in prison (1 = yes)	0.07	0.15	0.25 *	0.09	−0.17	0.06	0.12	0.04	−0.08	−0.00	0.10	0.26 **	−	0.04	0.06	0.24 *	0.09	0.05
14. Letters	0.08	0.20 *	0.10	−0.01	−0.06	−0.04	−0.05	−0.03	0.14	0.18	−0.17	0.01	−0.02	−	0.09	0.0.01	0.16	0.05
15. Phone	0.17	0.30 **	−0.10	0.18	−0.10	−0.07	−0.02	−0.06	0.08	0.03	0.04	−0.11	0.23 *	0.16	−	0.34 **	−0.14	0.47 **
16. Visits	0.24 *	0.20*	−0.10	0.18	−0.19	0.03	−0.05	−0.09	0.01	−0.0.00	−0.09	−0.13	0.29 **	0.37 **	0.40 **	−	−0.11	0.20 *
17. Figure of caregiver (1 = other parent)	0.07	−0.09	0.03	0.07	0.08	0.01	0.14	−0.04	0.08	−0.06	−0.08	−0.01	0.01	0.04	−0.05	0.15	−	−0.08
18. Nexus function	0.29 **	0.63 **	0.10	0.31 **	−0.13	0.11	−0.16	−0.01	0.05	−05	0.17	0.02	0.13	0.13	0.36 **	0.23 *	−0.08	−

* *p* < 0.05, ** *p* < 0.01. (men below the diagonal and women above the diagonal). Note: Pearson correlations were used between two continuous variables, point-biserial correlations between a categorical and a continuous variable, and phi coefficients between two categorical and dichotomous variables.

**Table 3:** Pearson correlations between the parenting experience (parental satisfaction and relationship with children) and the variables concerning the imprisoned parent, the children and the caregiver for Spaniards and foreigners.

	1	2	3	4	5	6	7	8	9	10	11	12	13	14	15	16	17	18
1. Parental satisfaction	−	0.50 **	−0.13	−0.14	0.12	0.13	−0.08	−0.12	−0.15	−0.13	0.16	0.03	0.21 *	0.05	−0.02	−0.07	0.02	0.11
2. Relationship with children	0.55 **	−	−0.01	−0.14	0.05	0.05	−0.10	−0.03	−0.18	−0.07	0.23 *	0.09	0.15	0.13	0.06	−0.00	0.01	0.37 **
3. Age	−0.05	−0.00	−	0.07	−0.11	−0.18	0.08	0.03	0.06	0.10	0.00	0.65 **	0.28 **	0.18	−0.21 *	0.02	0.10	−0.13
4. Partner (1 = yes)	0.34 **	0.38 **	−0.02	−	−0.14	0.04	−0.03	0.10	0.05	0.07	0.17	0.04	0.00	−0.00	−0.03	−0.04	0.12	0.05
5. Primary Education (1 = yes)	−0.08	−0.15	0.01	−0.20 *	−	0.03	−0.18	−0.06	−0.12	−0.04	−0.28 **	−0.14	−0.00	−0.21 *	0.07	−0.14	−0.07	−0.10
6. Penitentiary situation (1 = preventive)	0.12	0.20 *	−0.06	−0.01	0.16	−	−0.37 **	−0.12	−0.34 **	−0.29 **	0.08	−0.29 **	−0.02	0.13	−0.01	0.02	0.13	0.08
7. Time in prison	−0.15	−0.25 **	0.26 **	−0.04	0.03	−0.32 **	−	0.20*	0.30 **	0.23 *	−0.09	0.20 *	−0.11	−0.04	0.04	−0.02	−0.28 *	0.04
8. Furloughs	0.10	0.07	0.11	0.12	−0.05	−0.15	0.06	−	0.06	0.06	−0.06	0.14	−0.01	−0.07	0.04	0.12	−0.13	0.01
9. Paid job (1 = paid−employment)	−0.06	0.02	0.16	0.03	0.05	−0.16	0.20 *	−0.04	−	0.87 **	−0.13	0.13	−0.11	−0.06	0.09	0.01	−0.22 *	−0.05
10. Sent money to their children (1 = yes)	−0.19 *	0.02	0.11	0.03	0.06	−0.03	0.16	−0.11	−0.36 **	−	−0.05	0.18	−0.09	−0.10	0.06	0.00	−0.25 *	−0.02
11. Number of children	0.09	0.07	−0.22 *	0.19 *	−0.23 *	0.07	−0.05	−0.02	−0.12	−0.04	−	0.01	0.13	0.21 *	−0.12	−0.13	0.10	0.05
12. Age of children	0.09	0.08	0.53 **	−0.06	−0.15	−0.04	0.24 *	0.16	0.04	−0.08	−0.08	−	0.38 **	0.03	−0.14	0.00	−0.01	−0.10
13. Children know parent is in prison (1 = yes)	0.16	0.27 **	0.19 *	0.24 *	−0.28 **	0.06	0.10	0.06	0.01	0.01	0.20 *	0.38 **	−	0.23 *	0.03	0.10	0.02	−0.03
14. Letters	0.18	0.23 *	0.03	0.11	0.02	−0.00	−0.09	−0.06	0.11	0.19 *	−0.09	−0.04	−0.13	−	−0.02	−0.02	0.09	−0.01
15. Phone	0.34 **	0.49 **	−0.07	0.34 **	−0.03	0.09	−0.10	0.06	0.17	0.01	0.20 *	−0.14	0.23 *	0.19	−	0.14	−0.20	0.25 *
16. Visits	0.20 *	0.33 **	−0.14	0.29 **	−0.07	0.13	−0.08	0.04	0.13	−0.03	0.05	0.02	0.27 **	0.16	0.48 **	−	−0.13	0.02
17. Figure of caregiver (1 = other parent)	−0.02	−0.13	0.12	−0.02	0.13	−0.09	0.25 *	−0.24 *	0.15	0.10	−0.08	−0.09	−0.01	0.12	−0.08	−0.02	−	−0.18
18. Nexus function	0.32 **	0.72 **	0.05	0.41 **	−0.08	0.15	−0.10	0.17	0.05	0.05	0.10	0.09	0.26 **	0.18	0.56 **	0.39 **	−0.01	−

* *p* < 0.05, ** *p* < 0.01. (Spaniards below the diagonal and foreigners above the diagonal). Note: Pearson correlations were used between two continuous variables, point-biserial correlations between a categorical and a continuous variable, and phi coefficients between two categorical and dichotomous variables.

**Table 4:** Hierarchical multiple regression with parental satisfaction as criterion variable.

	Model 1	Model 2	Model 3	Model 4	Model 5	Model 6	Final Model
	*B* *(SE)*	*B* *(SE)*	*B* *(SE)*	*B* *(SE)*	*B* *(SE)*	*B* *(SE)*	*B* *(SE)*
Block 1: Sociodemographics							
Partner (1 = yes)	0.098 (0.052)	0.102 (0.052)	0.066 (0.053)	0.038 (0.052)	0.052 (0.053)	0.183 * (0.075)	0.201 ** (0.072)
Block 2: Penitentiary variables							
Sent money to children (1 = yes)		−0.134 * (0.062)	−0.132 * (0.061)	−0.141 * (0.060)	−0.161 * (0.061)	−0.238 ** (0.088)	−0.150 * (0.060)
Block 3: Children							
Children know parent is in prison (1 = yes)			0.091 (0.053)	0.090 (0.052)	0.096 (0.052)	−0.010 (0.085)	
Frequency of letters			0.001 (0.001)	0.001 (0.001)	0.002 (0.001)	0.001 (0.001)	
Frequency of phone calls			0.001 * (0.001)	0.001 (0.001)	0.001 (0.001)	0.001 (0.001)	
Frequency of visits			−0.001 (0.003)	−0.001 (0.003)	0.001 (0.003)	0.004 (0.005)	
Block 4: Primary Caregiver							
Nexus function				0.070 ** (0.023)	0.059 * (0.024)	0.051 * (0.024)	0.067 ** (0.022)
Block 5: Moderators							
Sex (1 = women)					0.046 (0.050)	0.082 (0.092)	
Nationality (1 = foreigner)					0.078 (0.058)	0.196 (0.147)	−0.077 (0.064)
Block 6: Interactions							
Partner*Nationality						0.274 ** (0.102)	0.280 ** (0.099)
Sent money*Nationality						−0.162 (0.118)	
Children know*Sex						−0.142 (0.099)	
Children know*Nationality						−0.066 (0.104)	
Freq. of phone calls*Sex						0.001 (0.001)	
Freq. of phone calls*Nationality						−0.001 (0.001)	
Freq. of visits*Sex						−0.008 (0.005)	
Freq. of visits*Nationality						−0.018 (0.022)	
Freq. of letters*Sex						0.001 (0.001)	
Δ*R*^2^ *R*^2^	0.017	0.022 * 0.040	0.057 * 0.097	0.042 ** 0.139	0.012 0.151	0.075 * 0.227	0.035 ** 0.146

* *p* < 0.05, ** *p* < 0.01.

**Table 5:** Hierarchical multiple regression with relationship with children as criterion variable.

	Model 1	Model 2	Model 3	Model 4	Model 5	Model 6	Final Model
	*B* *(SE)*	*B* *(SE)*	*B* *(SE)*	*B* *(SE)*	*B* *(SE)*	*B* *(SE)*	*B* *(SE)*
Block 1: Sociodemographics							
Partner (1 = yes)	0.156 * (0.068)	0.146 * (0.067)	0.058 (0.066)	−0.026 (0.056)	−0.003 (0.056)	0.080 (0.089)	0.192 * (0.077)
Block 2: Penitentiary variables							
Penitentiary situation (1 = preventive)		0.102 (0.088)	0.077 (0.083)	0.006 (0.070)	0.015 0.069	0.091 (0.080)	
Time in prison		−0.002 ** (0.001)	−0.002 ** (0.001)	−0.002 ** (0.001)	−0.002 * (0.001)	−0.001 (0.001)	
Furloughs		0.005 (0.010)	0.005 (0.010)	−0.001 (0.008)	−0.004 (0.008)	−0.047 * (0.020)	−0.057 ** (0.020)
Block 3: Children							
Number of children			0.026 (0.029)	0.022 (0.024)	0.022 (0.024)	−0.034 (0.033)	−0.011 (0.032)
Children know parent is in prison (1 = yes)			0.133 * (0.066)	0.129 * (0.055)	0.125 * (0.055)	0.122 (0.090)	
Frequency of letters			0.002 (0.001)	0.002 (0.001)	0.002 * (0.001)	0.002 (0.001)	
Frequency of phone calls			0.002 *** (0.000)	0.001 (0.000)	0.001 (0.000)	0.001 (0.001)	
Frequency of visits			0.002 (0.004)	0.001 (0.003)	0.002 (0.003)	−0.003 (0.004)	
Block 4: Primary Caregiver							
Nexus function				0.225 *** (0.024)	0.220 *** (0.025)	0.200 *** (0.025)	0.229 *** (0.023)
Block 5: Moderators							
Sex (1 = women)					0.108 * (0.054)	0.182 (0.099)	0.120 * (0.054)
Nationality (1 = foreigner)					0.037 (0.060)	−0.220 (0.168)	−0.363 ** (0.124)
Block 6: Interactions							
Partner*Sex						−0.147 (0.102)	
Partner*Nationality						0.300 * (0.108)	0.329 ** (0.106)
Penitentiary situation*Nationality						−0.172 (0.125)	
Time in prison*Sex						0.000 (0.001)	
Time in prison*Nationality						−0.001 (0.002)	
Furloughs*Sex						−0.050 * (0.022)	0.058 ** (0.022)
Number of children*Nationality						0.117 * (0.050)	0.110 * (0.048)
Children know*Sex						−0.071 (0.102)	
Children know*Nationality						0.086 (0.108)	
Freq. of letters*Sex						0.001 (0.002)	
Freq. of letters*Nationality						−0.001 (0.002)	
Freq. of phone calls*Nationality						−0.001 (0.001)	
Freq. of visits*Nationality						0.001 (0.023)	
Δ*R*^2^ *R*2	0.026 *	0.060 ** 0.086	0.126 *** 0.211	0.248 *** 0.459	0.012 0.472	0.065 * 0.536	0.054 *** 0.472

* *p* < 0.05, ** *p* < 0.01, *** *p* < 0.001.

## Data Availability

The data presented in this study are available on request from the corresponding author. The data are not publicly available due to assure the data protection stemming from a vulnerable population.
